# A Study on the Characteristics of the Ionospheric Gradient under Geomagnetic Perturbations

**DOI:** 10.3390/s20071805

**Published:** 2020-03-25

**Authors:** Yixin Zhang, Yang Liu, Junlei Mei, Chunxi Zhang, Jinling Wang

**Affiliations:** 1 School of Instrumentation and Opto-Electronic Engineering; Beihang University, Beijing 100191, China; yixinzhang@buaa.edu.cn (Y.Z.); meijunlei@buaa.edu.cn (J.M.); zhangchunxi@buaa.edu.cn (C.Z.); 2 School of Civil and Environmental Engineering, University of New South Wales, Sydney, NSW 2052, Australia; jinling.wang@unsw.edu.au

**Keywords:** ionospheric gradient, geomagnetic storm, sigma bound, ground-based augmentation system, satellite navigation

## Abstract

The Earth’s ionosphere is greatly influenced by geomagnetic activities, especially geomagnetic storms. During a geomagnetic storm, the ionosphere suffers many perturbations, leading to a spatial gradient that are neglected during geomagnetically quiet periods. An ionospheric gradient generates potential hazards for a ground-based argumentation system (GBAS) by enlarging the errors in the delay corrections between ground monitor stations and users. To address this problem, this work investigates the characteristics of the ionospheric gradient under geomagnetic storms. Global Navigation Satellite System (GNSS) observations from the continuously operating reference station (CORS) network were used to analyze the ionospheric gradients during the geomagnetic storm on 8 September 2017. The statistical behavior of the ionospheric gradient was further discussed. Experiments show that strong geomagnetic perturbations lead to large ionospheric gradients, and the gradients also vary with the geomagnetic location.

## 1. Introduction

The ionosphere is a portion of the upper atmosphere ranging from 60 to 1000 km above the Earth’s surface. The atoms in the ionosphere are ionized by ultraviolet radiation from solar heating and X radiation during solar flares [1,2]. Hence, the ionosphere has a critical impact on trans-ionosphere signals, especially GNSS signals [3]. Due to signal refraction, the ionosphere produces a delay for the GNSS signals, leading to ranging error [4]. A differential GNSS uses error corrections from monitor stations to eliminate the ionospheric delay. This strategy works only under the assumption of consistency of the ionospheric delay between the monitor stations and users. In a GBAS, the performance of the ionospheric delay correction is evaluated by the standard deviation of the ionospheric gradient [5].

The ionospheric gradient refers to the spatial difference in the ionospheric range delay [6]. Usually, the spatial ionospheric gradient is 1 mm/km, and one sigma bound is less than 4 mm/km [7,8]. However, the ionosphere is closely influenced by solar and geomagnetic activities [9,10,11,12]. The ionospheric gradient under high solar activity can be ten times that under low solar activity; moreover, during geomagnetic disturbances and geomagnetic storms, the response of the ionosphere can generate a large ionospheric gradient, leading to immense spatial decorrelations of the ionospheric delay error [13,14]. A noticeable ionospheric gradient hazard was observed during a geomagnetic storm in November 2003 [15,16]. The largest ionospheric gradient in America was 412 mm/km, causing a ranging error of 8.4 m, enough to threaten the GBAS precision landing performance [17]. At low latitudes, the largest ionospheric gradient was discovered to be approximately 850 mm/km in Brazil [18,19]. In Asia, a 138.5 mm/km gradient was observed which had been induced by a medium-scale traveling ionospheric disturbance (MSTID) on 10 November 2004 [20]. An extremely large gradient of 540 mm/km was discovered due to the activity of plasma bubbles in Thailand, and gradients above 300 mm/km were also observed at low latitudes in Hong Kong and Singapore, where plasma bubbles are easily generated [21,22]. A similar study of the June 2015 storm also revealed a close association between a large gradient and ionospheric irregularities [12]. Previous results revealed that ionospheric gradients have daily and seasonal variabilities and show dominant activity during equinoctial months [23]. The ionospheric gradient at Suvarnabhumi Airport shows that, at low latitudes, the ionospheric gradient can vary from 28 to 178 mm/km. Another study of the behaviors of the ionospheric gradient under quiet conditions was conducted in the same airport and revealed that the background ionospheric gradient at low latitudes was less than 10 mm/km but still fluctuated from equinoctial months to solstice months [24]. 

For existing studies, the CORS network has densely distributed GNSS observatories in a regional scope, providing sufficient observables to investigate the ionospheric gradient at a small spatial scale [25]. More experiments have suggested that the ionospheric gradient is much smaller over a smaller region than over a larger region, for example, the ionospheric gradient in America is often larger than that in South Korea [26]. This variability in the ionospheric gradient is associated with several influential factors, especially the geographical location and solar and geomagnetic activities, and thus the one sigma bound should be carefully selected to cover all potential extreme gradients which is critical for guaranteeing the GBAS precision approaching and landing performance [27]. The bounding Gaussian distribution has been used to describe the extreme delay error induced by ionosphere disturbance, and the method has been accepted in GBAS studies [28,29]. A surly, ionospheric reaction to geomagnetic disturbances has varied responses, such as an increase or decrease of total electron content (TEC), enhancement of the rate of the TEC index (ROTI), traveling ionospheric disturbance, as well as other variabilities observed by the ionosonde [11,12,30,31]. The focus of this method is to evaluate ionospheric gradients from the aspect of GBAS studies, to see how much error ionospheric disturbances can bring to the spatial inconsistence of delay error, which is important for the ionospheric hazard in GBAS [32]. 

An intense geomagnetic storm occurred on 8 September 2017. In this work, the statistical behavior of the ionospheric gradient during this storm was studied. CORS data from America, Italy, Australia, and New Zealand were selected to derive the corresponding ionospheric gradients. To supplement this analysis, the features under quiet geomagnetic conditions were also considered for investigation.

Section 2 describes data and methodology used in this work, Section 3 outlines experimental results, Section 4 provides discussions about the results, followed by the concluding remarks. 

## 2. Data and Methodology 

### 2.1. Data Representation

**Figure 1:**
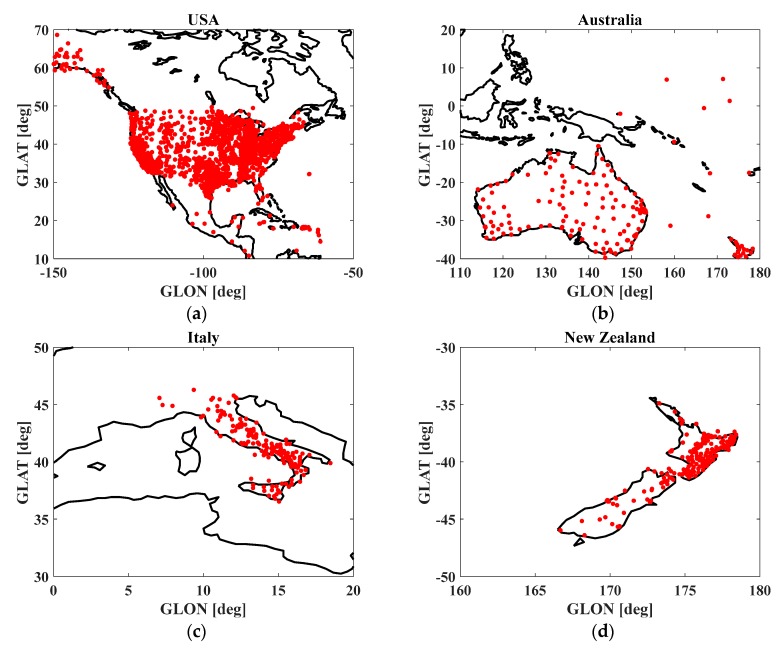
Continuously operating reference station (CORS) networks that provided the data used in this work. (**a**) USA CORS network; (**b**) Australia CORS network; (**c**) Italy CORS network; (**d**) New Zealand CORS network.

The CORS network provides GNSS dual-frequency observables in a receiver independent exchange(RINEX) format, and thus the ionospheric TEC can be calculated. The geographical distribution of the data is demonstrated in Figure 1. The CORS network in the USA provides over 1800 stations for observables spread throughout the continent, United States (CONUS) and Alaska. The networks in Italy, Australia, and New Zealand provide a combined total of over 800 stations for observables. To calculate the ionospheric gradients, stations with separation distances of less than 35 km were selected as pairs, thus, not all CORS observables were used for this analysis. To study the geomagnetic perturbations of this storm, geomagnetic indices such as Dst and AE were collected from the International Service of Geomagnetic Indices (ISGI). Days of geomagnetic quiescence were also provided by the ISGI; here, three quiet days in August 2017 were considered for analysis. The interplanetary magnetic field (IMF) was provided by Goddard Space Physics Flight Center (https://omniweb.gsfc.nasa.gov/) to judge the commencement of storm. 

### 2.2. Calculation of the TEC and the Ionospheric Gradient

Under the thin-shell ionosphere assumption, a trans-ionosphere propagating signal intersects a thin shell where electrons and ions are concentrated; this intersection point is considered the ionospheric pierce point (IPP). The slant TEC (STEC) at the IPP can be derived from the satellite delay, and then transferred to the vertical TEC (VTEC) by a mapping function. The mapping function is defined by Equation (1). Multiplying the STEC by Equation (1) gives the vertical TEC in Equation (2). In Equation (2), α denotes the zenith angle from the satellite to the receiver, R_e_ is the Earth’s radius, set to 6371 km, and H denotes the altitude of the thin shell from the Earth’s surface, set to 350 km in this work. Both the STEC and the VTEC are in TECU (1 TECU is 10^16^ electrons). The proposed TEC calculation is in reference to Ciraolo’s arc-offset method and realized on the software platform developed by the T/ICT4D Lab of International Centre for Theoretical Physics [33]. The ionospheric delay in relation to the TEC is given as Equation (1):

(1) $$\mathrm{STEC}=\frac{f_{1}^{2}}{40.3}I$$

where I denotes the ionospheric delay in metres and f_1_ represents the L1-band frequency of GPS signals. One TECU is related to a delay of 0.16 m for ranging. The slant TEC can be converted into the vertical TEC by a mapping function:

(2) $$\mathrm{MF}\left(\alpha \right)={\left[1-{\left(\frac{R_{e}\cos\alpha }{R_{e}+H}\right)}^{2}\right]}^{1/2}$$

where α denotes the elevation angle (above 30 degrees in this work to eliminate multipath interference).

(3) VTEC=STEC×MF(α)

The ionospheric gradient is calculated by dividing the distance between two stations by the difference in the TEC between these two stations and is given as:

(4) $$I_{g}=\frac{VTEC_{1}-VTEC_{2}}{d_{IPP}}\cdot \frac{40.3}{f_{L1}^{2}}\left[\mathrm{mm}/\mathrm{km}\right]$$

where $d_{IPP}$ is the distance between the two stations, also considered as the baseline distance and $I_{g}$ is the ionospheric gradient (in units of mm/km) representing the ranging delay caused by TEC difference across every unit distance. $I_{g}$ can reflect both the spatial variation in the ionosphere and the influence of the ranging error on navigation [12,23]. 

### 2.3. One Sigma Bound of the Ionospheric Gradient

The ionospheric gradient greatly impacts the differential correction of the GBAS ranging error. To assess this impact, the parameter $\sigma_{\mathrm{overbound}}$ is introduced, here, that mainly considers the statistics of long-term ionospheric gradient observables [34]. The idea for this parameter first comes from the principles of precision approaching under a local area augmentation system (LAAS), a GBAS designed by the USA [17]. The variability of the vertical ranging error induced by the ionospheric gradient is evaluated. The users can assess the impacts of the ionospheric gradient by the standard deviation, $\sigma_{\mathrm{vig}}$. The proposed parameter is calculated under the assumption that the delay error distribution follows a Gaussian distribution, which is not true in reality. In general, the standard Gaussian distribution cannot cover all the errors induced by ionospheric gradients. Extreme values exist in the tail of the Gaussian probability density function (PDF), known as the “heavy tail” feature. A reliable way to solve this problem is to bound the PDF at the tails. The parameter $\sigma_{\mathrm{overbound}}$ based on the Gaussian distribution relies on a certain hazard probability. After the computation of I_g_, the data are evenly divided into several parts with the same time interval. For all the observables from different stations, each part with the same time interval is assembled to derive $\sigma_{\mathrm{overbound}}$ and evaluate the TEC [35]. The calculation of $\sigma_{\mathrm{overbound}}$ is given as:

(5) $$\sigma_{\mathrm{overbound}}=\left|\mu_{\mathrm{vig}}\right|+f\sigma_{\mathrm{vig}}$$

where $\sigma_{\mathrm{vig}}$ and $\mu_{\mathrm{vig}}$ denote the standard deviation and mean value, respectively, and f is the bounding factor. The results are then normalized as:

(6) $$\bar{I_{g}}=\frac{I_{g}-\mu_{\mathrm{vig}}}{\sigma_{\mathrm{vig}}}$$

The probability density function is fitted by a Gaussian distribution; then, the standard deviation of the Gaussian distribution is inflated to obtain a new distribution until all the ionospheric gradients are bounded. Here, the inflation factor starts from 1 and increases by a step of 0.5. When the satisfied distribution is achieved, the inflation factor increases by a step of 0.05 until all the ionospheric gradients are well bounded.

**Figure 2:**
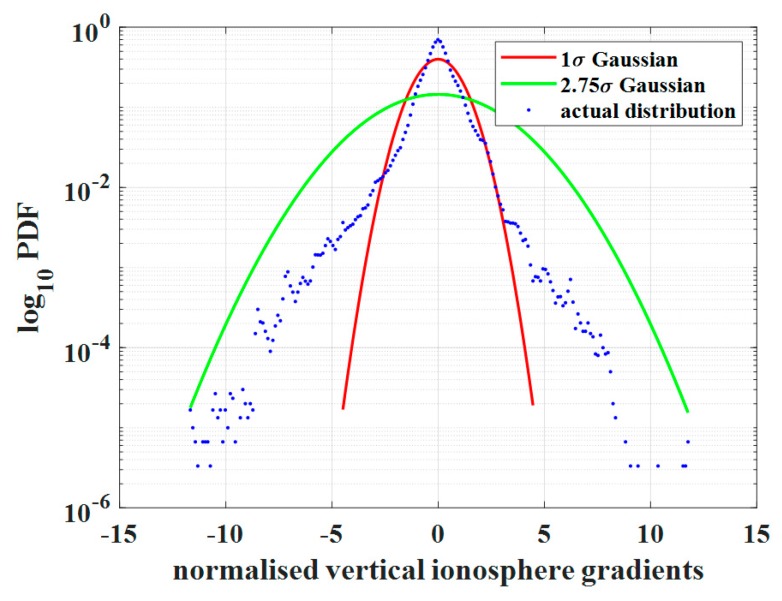
Fitting and bounding Gaussian distributions of the ionospheric gradient; the results were derived from the CORS data used in this work. The red curve indicates the 1 σ Gaussian distribution probability density function (PDF), the green curve indicates the bounded Gaussian distribution PDF with an inflation factor of 2.75, and the blue dots are the real gradients derived from observables.

Figure 2 shows the fitting Gaussian distribution and bounding Gaussian distribution. The blue dots demonstrate the real distribution of the ionospheric gradient, the red line is the fitting distribution curve, and the green line is the new distribution after inflating the standard deviation. As this figure shows, the inflated Gaussian distribution can cover the probability distribution of all large ionospheric gradients. Here, the inflation factor is 2.75. The amount of this inflation factor is proportionally determined by the amount of the existing extreme ionospheric gradient.

## 3. Experimental Results

The geomagnetic storm that occurred on 8 September 2017 was selected as a case study for the response of the ionospheric gradient, and the results were compared with the response on geomagnetically quiet days. The American CORS network was mainly considered; pairs of stations with separation distances of less than 35 km were selected. Other stations from CORS networks in Australia, Italy, and New Zealand were also considered, and the results were compared.

### 3.1. General Morphology of the Geomagnetic Storm

**Figure 3:**
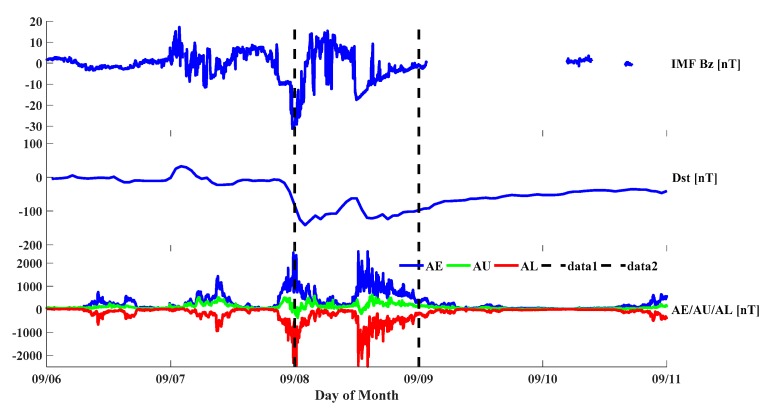
General morphology of the geomagnetic storm on 8 September 2017 (corresponding to day 251), the three panels plot interplanetary magnetic field (IMF) Bz, the Dst and AE, AU, and AL indices. All those geomagnetic indices use the unit of nT. For the third panel, the blue, green, and red curves indicate AE, AU and AL indices.

A strong geomagnetic storm struck on 7 September 2017 and lasted two days, as shown in Figure 3. To study this geomagnetic storm, the corresponding geomagnetically quiet days were selected within the month, with international days of geomagnetic quiescence as references. From the ISGI reports, they were selected from 8 to 10 August 2017. The IMF Bz is used to show the commencement of geomagnetic storm, when its value suddenly drops and reaches minus ten to twenty nT. The Dst index takes the average of the horizontal geomagnetic components from the earth around equatorial region and indicates the intensity of a geomagnetic storm. When a geomagnetic storm occurs, the Dst index quickly drops below −50 nT, and even below −100 nT if it is a strong geomagnetic storm. The AE index denotes the initiation of the auroral electrojet and measures the auroral zone magnetic activity produced by enhanced ionospheric currents flowing below and within the auroral oval. The AE index is calculated by the largest and smallest values of geomagnetic variations in horizontal component, which are observed at selected (10 to 13) observatories along the auroral zone in the Northern Hemisphere. The AU and AL indices are defined by the above largest and smallest values, respectively, and the difference between AU and AL defines the AE index.

On 7 September, the IMF Bz turned southward and quickly dropped to below −20 nT, indicating the first subphase of the geomagnetic storm. This period lasted from midnight to 03:00 UT on 8 September. The AE index increased drastically with perturbations during this period; the maximum AE value was reached above 2000 nT, and then the AE gradually decreased with oscillations over the next several hours. At 12:00 UT on 8 September, the Dst index exhibited another minor decrease, accompanied by a second phase of enhancements and perturbations in the AE index that lasted until 18:00 UT on the same day. The maximum AE index was reached above 2000 nT, after which AE decreased with perturbations. During these periods, multiple scales of ionospheric responses occurred, and tremendous TEC variabilities were noticed in the USA, leading to the further perturbation of the ionospheric gradient.

### 3.2. Responses of the Ionospheric Gradient

#### 3.2.1. Ionospheric Gradient under Geomagnetically Quiet Conditions

**Figure 4:**
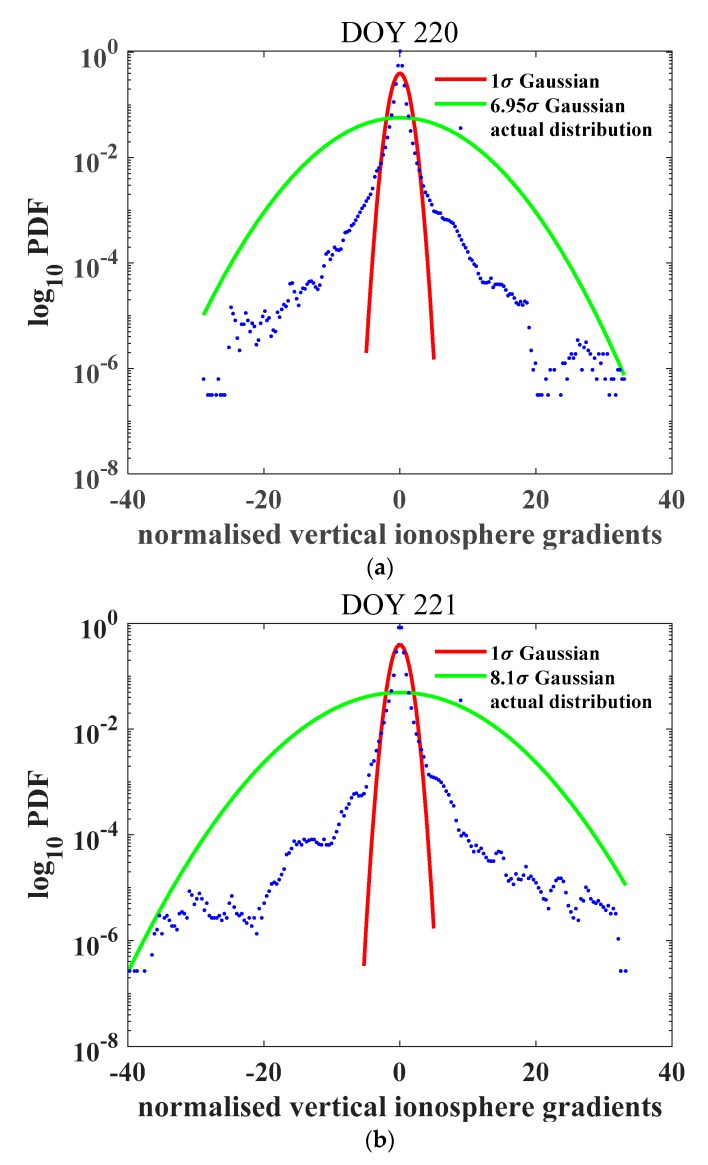
Probability density function of the ionospheric gradient derived during the geomagnetically quiet days. (**a**) 08–08 corresponding to day 220 of the year; and (**b**) 08–09 corresponding to day 221 of the year. The red curve indicates the 1σ Gaussian distribution PDF, the green curves indicate the bounded Gaussian distribution PDFs with inflation factors of 6.95 (a) and 8.1 (b), and the blue dots are the real gradients derived from observables. The inflation factors are different between the two geomagnetic quiet days.

The overbound of the standard deviation was calculated for the 8 and 10 August 2017, as shown in Figure 4. The normalized gradient distributions varied among the days. The bounded standard deviation values were 21.81 and 26.87 mm/km, separately, for the two days, corresponding to inflation factors of 6.95 and 8.1, respectively.

Then, the pairs of stations were grouped by their baseline distance to reveal the overbound of the standard deviation, as Table 1 shows. From the results for the three days, for most groups, the $\sigma_{\mathrm{overbound}}$ is within 10 mm/km. According to a comparison of the normalized gradient distributions for the pairs of stations with a 30 to 35 km baseline, a relatively small inflation factor occurred on 10 August 2017. However, the distributions for the stations with baseline distances of less than 10 km were quite different; all the inflation factors were large (exceeding 25 mm/km) and showed daily variations. These results show that the overbound of the standard deviation with a baseline distance of less than 10 km showed the most divergence as compared with the other groups and produced a considerable impact on the overall bounds. For the other baseline distances, the $\sigma_{\mathrm{overbound}}$ on the three days were close. For the baseline distances of 15 to 20 km, the smaller $\sigma_{\mathrm{overbound}}$ also occurred on 10 August. For the case of 30 to 35 km, $\sigma_{\mathrm{overbound}}$ on 10 August was also less than that on the previous day. The standard deviation of the ionospheric gradient, $\sigma_{\mathrm{vig}}$ decreased with increasing baseline distances. When the baseline distance was larger than 10 km, all the $\sigma_{\mathrm{vig}}$ observed were within 4 mm/km, which is similar to the cases from the past ten years [30]. The $\sigma_{\mathrm{vig}}$ under a 10 km baseline distance varied from 8.49 mm/km to 9.69 mm/km during geomagnetic quiet days of August.

#### 3.2.2. Ionospheric Gradient under the Geomagnetic Storm

**Figure 5:**
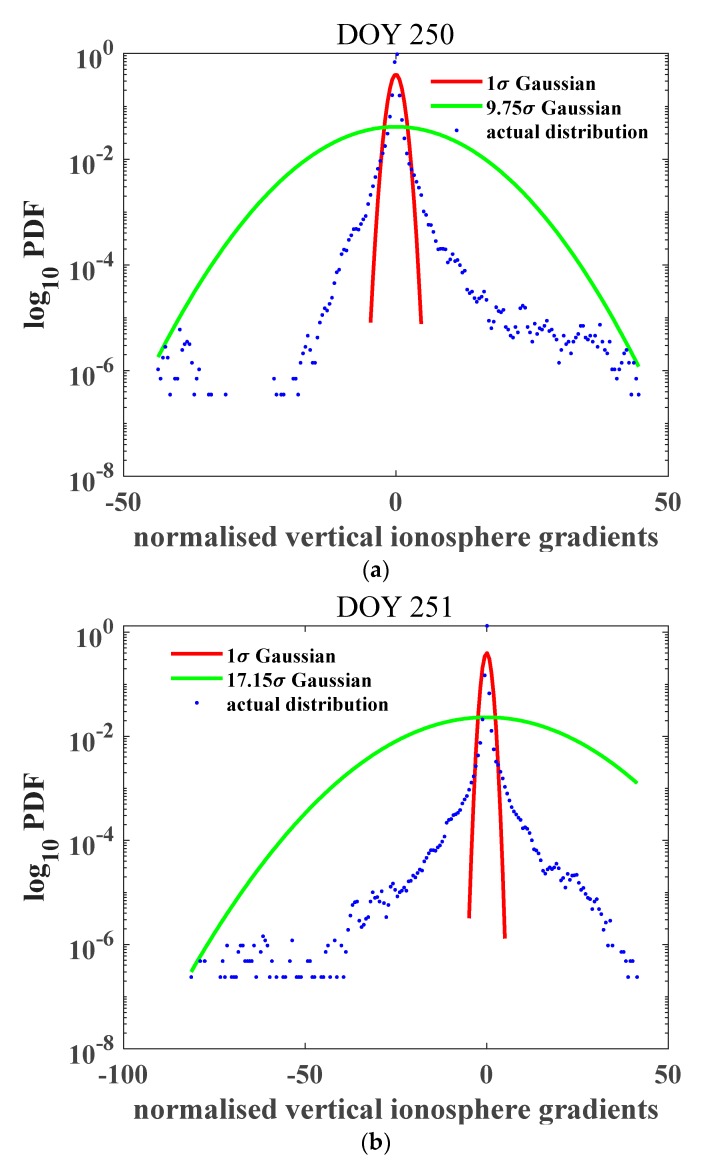
Probability density function of the ionospheric gradient derived during the geomagnetic storm period (**a**) 09–07 corresponding to day 250 of the year; and (**b**) 09–08 corresponding to day 251 of the year; the storm occurred at midnight on 8 September. The red curve indicates the 1σ Gaussian distribution PDF, the green curves indicate the bounded Gaussian distribution PDFs with inflation factors of 9.75 (a) and 17.15 (b), and the blue dots are the real gradients derived from observables. The inflation factor increased drastically due to the influence of the geomagnetic storm on 8 September.

$\sigma_{\mathrm{overbound}}$ was calculated for the two days during the geomagnetic storm. Prominent increases of 59.41 mm/km on 7 September and 283.91 mm/km on 8 September were observed; the inflation factors were 9.75 and 17.15, respectively, for the two days. Figure 5 represents the normalized gradient distribution. The ionospheric gradient increased drastically on the storm day, corresponding to the large gradients observed from the CORS network. A probable explanation for this feature is the strong ionospheric perturbations during the geomagnetic storm; the ionospheric irregularities generated by the prompt penetration electric field during the geomagnetic storm are also responsible.

**Figure 6:**
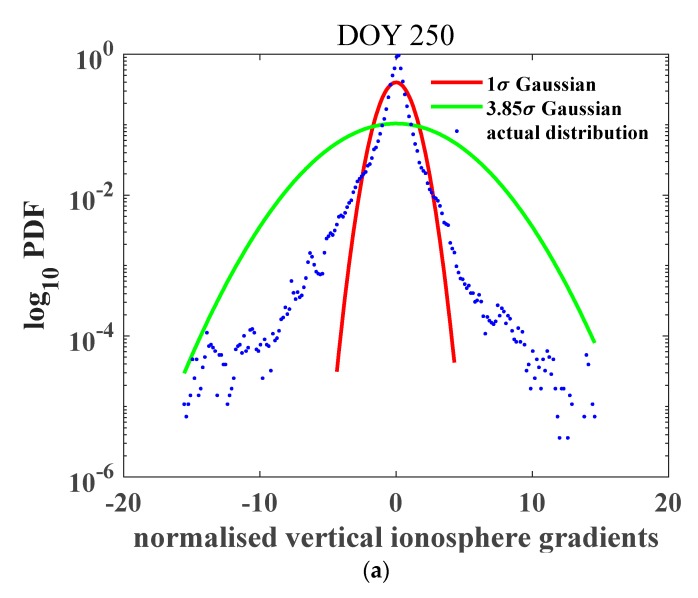

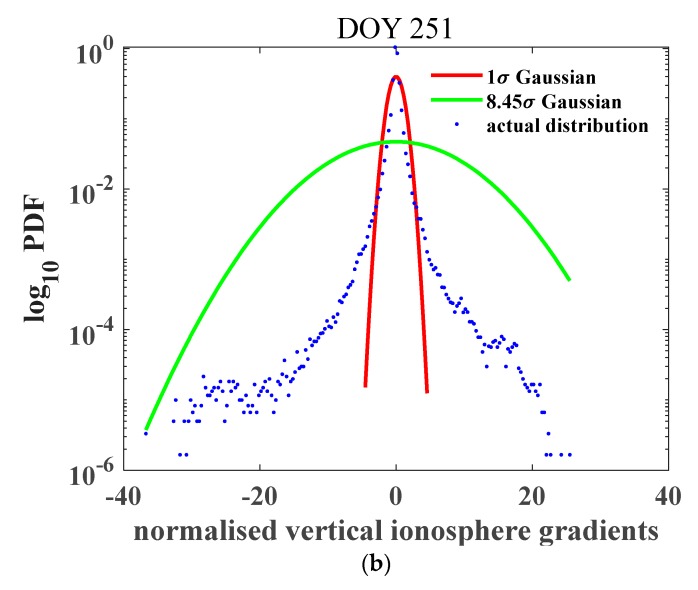
Probability density function of the ionospheric gradient derived during the geomagnetic storm period. (**a**) 09–07 corresponding to day 250 of the year; and (**b**) 09–08 corresponding to day 251 of the year; the storm occurred at midnight on 8 September, and the baseline distance range was from 30 to 35 km). The red curve indicates the 1σ Gaussian distribution PDF, the green curves indicate the bounded Gaussian distribution PDFs with inflation factors of 9.75 (a) and 17.15 (b), and the blue dots are the real gradients derived from observables. The inflation factor increased drastically due to the influence of the geomagnetic storm on 8 September.

The impact of the baseline distance was further considered, as demonstrated in Table 2. From the results on these two days, the overbound values on 8 September all exceeded the values on 7 September with larger inflation factors. The values for pairs of stations with baseline distances within 10 km increased to 325.31 mm/km on 8 September, over four times the value on the previous day. It was enhanced in proportion to the intensity of the geomagnetic perturbation. The $\sigma_{\mathrm{vig}}$ decreased with increasing baseline distances on both 7 September and 8, but the values exceeded 4 mm/km when the baseline distance was less than 25 km before the storm day, and the values of $\sigma_{\mathrm{vig}}$ under 10 km increased to 15.37 mm/km before the storm day, further reached to 50.41 mm/km on the storm day. Figure 6 represents the normalized gradient distribution, the baseline distance was 30–35 km. The inflation factor increased greatly from 3.85 in doy 250 to 8.45 in doy 251, indicating that more extreme values exist during the storm day. However, compared with Figure 5 the inflation factors were smaller, due to the selection of the longest baseline distance group considered in this work. 

Most CORS stations in America are in the geomagnetic middle latitudes and the sub-auroral region, with minor stations located around the auroral region at low latitudes. After grouping the paired stations, most pairs with distances larger than 10 km are in the middle latitudes and sub-auroral region, while only one to two pairs were in the auroral region. All paired stations were divided into the following three groups: those in the auroral region, the sub-auroral region, and the middle latitudes. The results are listed in Table 3 and Table 4. In Table 3, the overbound and standard deviation on the geomagnetically quiet days are shown. As the results show, the values decreased from the auroral region to the middle latitudes when the paired stations were separated by less than 10 km. The largest value occurred on 10 August, reaching 70.32 mm/km in the auroral region; the smallest value occurred on the same day in the middle latitudes with a value of 13.38 mm/km. For the sub-auroral region, the overbound value decreased with increasing distances; for instance, on 8 August, it dropped from 28.61 to 19.70 mm/km when the distance increased the range 10–15 km; on 10 August, it dropped tremendously from 36.81 to 16.64 mm/km when the distance increased to the range 10–15 km. Another sharp gap was observed in the sub-auroral region when the distance increased from the ranges 10–15 to 15–20 km; then, with increasing distance, the overbound value gradually decreased. Its features in the middle latitudes were slightly different because a slight increase in the overbound value was noticed when the distance increased from the range 10–15 to 15–20 km. On 8 August, the value increased from 5.08 to 5.84 mm/km, and on August 9, it greatly increased from 5.50 to 8.13 mm/km. A similar result was discovered on 10 August, but all the values were less than 10 mm/km when the distance was greater than 15 km in the middle latitudes. The standard deviation of the ionospheric gradient decreased when the distance increased to the range 10–15 km for both the sub-auroral region and middle latitudes on all three geomagnetic quiet days. The values were less than 4 mm/km when the distance increased greater than 5 km, consistent with a previous study for the USA middle latitudes. The standard deviation of the ionospheric gradients varied from 9.81 to 16.18 mm/km, and to 17.09 mm/km in the auroral region during the geomagnetic quiet days. When the distance was within 5 km, the standard deviation of the ionospheric gradient was larger in the middle latitudes than in the sub-auroral region. The minimum standard deviation of the ionospheric gradient in the middle latitudes was 7.76 mm/km on 10 August, and the maximum value in the middle latitudes was 8.66 on 8 August. The standard deviations of the ionospheric gradient in the sub-auroral region stayed below 7 mm/km during the geomagnetic quiet days. 

**Figure 7:**
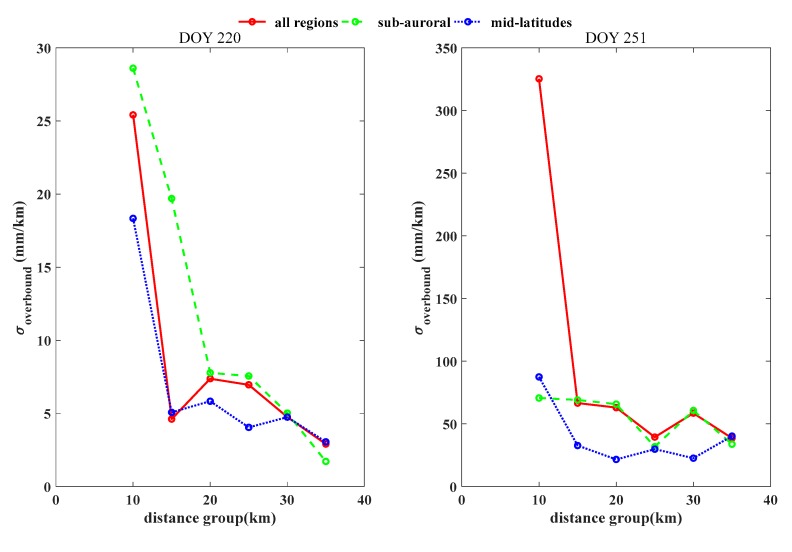
$\sigma_{\mathrm{overbound}}$ in relation to the baseline distance on 7 September and 8 September. The baseline distances are selected from 10 km to 35 km with 5 km intervals; the red, green, and blue dotted curves indicate the results for all regions, the sub-auroral region, and the middle latitudes, respectively. The overbound decreased greatly when the baseline distance was larger than 10 km. Extreme values over 300 mm/km were noticed on the storm day, 8 September.

The results in Table 4 show the overbound values during the geomagnetic storm. The values increased greatly in all the regions on 8 September, when the geomagnetic storm was dominant. The values in the auroral region exhibited the largest increment, reaching as high as 365.64 mm/km; however, contrary to the results during the days of geomagnetic quiescence, the values in the middle latitudes exceeded those in the sub-auroral region, indicating a strong disturbance of the ionospheric TEC in the middle latitudes under the geomagnetic storm, whereas the values in the sub-auroral region were also enhanced, although not as greatly. A probable explanation is that during the geomagnetic storm, a substantial TEC depletion was registered in the sub-auroral region, leading to an ionospheric trough. The values decreased gradually when the distance was larger than 15 km in the sub-auroral region (from 50.98 to 11.01 mm/km on 7 September), while overbound values fluctuated with increasing distances on 8 September, showing strong ionospheric randomness brought on by the geomagnetic storm. Similar results were discovered in the middle latitudes. On all days during the geomagnetic storm, the overbound values continued to follow latitudinal variations and decreased from the auroral region to the middle latitudes. The standard deviation of the ionospheric gradient increased drastically to 107.48 mm/km in the auroral region on 8 September, almost four times the value on the previous day. The standard deviation of the ionospheric gradient decreased when the distance increased to larger than 5 km. A fluctuation was noticed when the distances increased to the range 15–20 km, the value was 9.73 mm/km which was a slight increment as compared with that at 10–15 km in the sub-auroral region on 8 September. A similar feature was noticed in the middle latitudes when distances increased to the range 20–25 km, the value was 5.00 mm/km as compared with 4.87 mm/km at the 15 to 20 km distance, and 4.97 mm/km at the range 10–15 km. Figure 7 further demonstrates the dependence of baseline distance and the $\sigma_{\mathrm{overbound}}$, differnces of features were noticed between geomagnetic quiet day (doy 220) and the storm day (doy 251). The magnitude of $\sigma_{\mathrm{overbound}}$ increased greatly during the storm day, mostly contributed by the large $\sigma_{\mathrm{overbound}}$ in auroral region. The $\sigma_{\mathrm{overbound}}$ fluctuated with increment of the baseline distance after 15 km, and in general the magnitudes in midlatitudes were less than those in sub-auroral. For the geomagnetic quiet day, all the $\sigma_{\mathrm{overbound}}$ kept below 10 mm/km when the baseline distance was larger than 15 km. 

### 3.3. Ionospheric Variability in Different Regions

To conduct a more in-depth investigation, the ionospheric gradients in Italy, Australia, and New Zealand were calculated during the same period. Paired stations separated by distances from 5 to 20 km were selected to derive the overbounds. Two geomagnetically quiet days (8 August and 9) and two days of geomagnetic disturbance (7 September and 8) were considered for the comparison, the results were shown in Table 5. America had the largest ionospheric gradient on the storm day, reaching 148.20 mm/km, while New Zealand showed the smallest response at only 20.79 mm/km. In general, the impacts of this geomagnetic storm on the ionospheric gradient were mainly prominent in the Northern Hemisphere, especially the American continent. The standard deviation of the ionospheric gradient was dominant in USA during the geomagnetic storm as compared with Australia, Italy, and New Zealand. The minimum value was noticed in New Zealand, only 4.17 mm/km on 8 September; the maximum value was noticed in USA, 19.12 mm/km as an average value on 8 September. The temporal variations of regional behaviors for overbound and standard deviation were further investigated to see the perturbations of ionospheric gradients during geomagnetic storm on 8 September.

**Figure 8:**
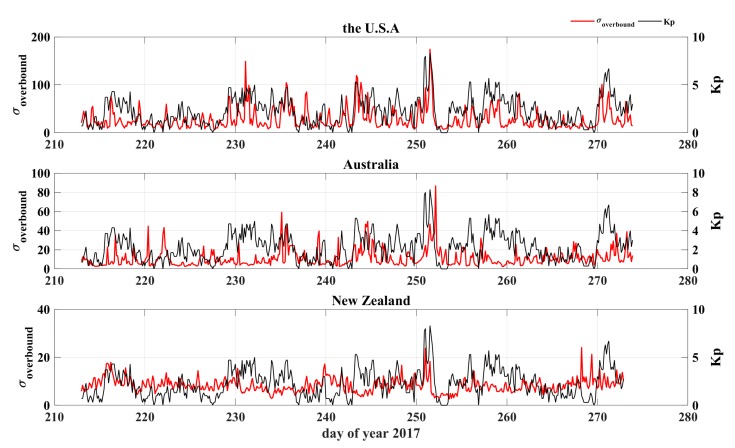
The overbound variability in relation to the Kp index for two-month durations (from 1 August to 30 September 2017). The red line indicates the overbound, and the black line indicates the Kp index. The results for the USA, Australia, and New Zealand are demonstrated. Large values correspond to large geomagnetic perturbations.

The long-term variation in the ionospheric gradient was considered next. Two months in 2017 were chosen for analysis. The CORS data from America, Australia, and New Zealand were used with pairs of stations separated by distances ranging from 5 to 20 km as candidates. The overbound was computed every three hours in relation to the Kp index for the two months to better represent the relationship between the geomagnetic disturbance and the TEC gradient. The results in Figure 8 show that a large overbound value corresponds to strong geomagnetic perturbations, indicated by the variability of the Kp index. However, slight variations were observed during the geomagnetically quiet period in Australia. The overbound values in the USA were more sensitive to the geomagnetic perturbations, with the maximum value exceeding 150 mm/km, while those in New Zealand were the least sensitive (all the values were below 25 mm/km).

## 4. Discussion

For those pairs with distances of less than 10 km, the large overbound values are the first concern for hazards to the navigation integrity, especially the hazards located in the auroral region. However, in general, the overbound value decreased from the auroral region to the middle latitudes based on the results from the USA. The overbound values showed the least variability in the sub-auroral region with an increasing paired station distance during the geomagnetically quiet period. When the baseline distance ranged from 15 to 35 km, the overbound values for all the USA were less than 10 mm/km for all the geomagnetic quiet days considered. For the same baseline distance, the overbound values show decrements from the sub-auroral region to the middle latitudes for most cases. Some exceptions were discovered in which the overbound values of the middle latitudes exceeded the value of the sub-auroral region. A probable explanation is that statistical randomity exists in the overbound to generate the inconsistences of value decrement, with increasing baseline distances. This means the overbound value depends on both the statistical nature of ionospheric gradient and the inflation factor calculation. The standard deviation of the ionospheric gradient decreased with increasing baseline distances in the sub-auroral region and the middle latitudes for all the geomagnetic quiet days. When the baseline distance was larger than 10 km, the standard deviations of the ionospheric gradients were less than 4 mm/km during geomagnetic quiet days, showing consistency with previous studies [7,8].

During the geomagnetic storm, the overbound value increased drastically, and the results in the auroral region were dominant. However, the values in the middle latitudes showed great variability with increasing baseline distance, indicating the variability of the ionospheric TEC in the middle latitudes during the geomagnetic storm. It was noticed that at the baseline distance from 10 to 30 km, the overbound values showed a decrement from the high to the middle latitudes. Larger overbound values were discovered in the middle latitudes than the sub-auroral region for the baseline ranges 5–10 km and 30–35 km during the storm day. Again, it was addressed by the statistical randomity of the overbound calculation, for which the inflation factor also has an influence. A large inflation factor indicates higher randomity and uncertainty. The standard deviation of the ionospheric gradient during the storm day showed fluctuations with increasing baseline distances, and it was caused by the ionospheric responses during the geomagnetic storm. 

The storm-induced prompt penetration of the electric field (PPEF) can produce ionospheric irregularities that propagate from the high latitudes to the middle latitudes, as validated by [36,37,38,39]. Ionospheric irregularities have been responsible for a large ionospheric gradient [12,19], leading to the strong divergence of overbound values in relation to the baseline distance. The fluctuations of overbounds and standard deviation of the ionospheric gradient in the sub-auroral reigon and the middle latitudes were mostly attributed to the ununiform ionosphere electron density excited by the geomagnetic storm, mainly the PPEF effect. 

The maximum ionospheric gradient during the geomagnetic storm reached 980 mm/km when the baseline distance was between 25 and 30 km at 01:07 UT; when the baseline distance varied from 10 to 35 km, the maximum ionospheric gradients were 685.02 mm/km at 00:31 UT, 767.8 mm/km at 00:53 UT, 834 mm/km at 00:45 UT, 980 mm/km at 01:07 UT, and 918.95 mm/km at 00:51 UT, with 5 km as the distance range span. In addition, for all the maximum ionospheric gradient arrays from each pair sites, more than 71% cases were concentrated in the period before 03:00 UT, when the main phase of geomagnetic storm was dominant. All the values were larger than that ever recorded in America, i.e., 412 mm/km [17]. Those large ionospheric gradients from 25 to 30 km and 30 to 35 km exceeded previous result from Brazil low latitudes [18,19]. All the large values were discovered in the main phase of the storm. 

The results from Italy, Australia, and New Zealand were consistent with the behaviors of the ionospheric gradients during the geomagnetic storm. However, from the increment magnitude, America was the most sensitive to geomagnetic perturbations, followed by Italy, indicating longitudinally and hemispherically asymmetric responses to the geomagnetic storm. A strange inconsistency was observed in Australia and New Zealand, where slight geomagnetic perturbations also occurred during geomagnetically quiet days, which was unusual as compared with results of low latitudes in Asia [24].

Another inconsistency with previous studies [29] was found for the standard deviation of the ionospheric gradient before the geomagnetic storm. According to Table 2, $\sigma_{\mathrm{vig}}$ showed variability with increasing baseline distances, the value calculated between 15 and 20 km was larger than that between 10 and 15 km. It was probably due to the combined effects of solar flux and geomagnetic disturbance, since an X-class solar flare had just happened on the previous day (6 September 2017). The geomagnetic disturbance was also attributed to the large and unusual $\sigma_{\mathrm{vig}}$ on 7 September as compared to the values in Table 1, which were computed in monthly geomagnetic quiet days. 

## 5. Conclusions

In this work, the features of the ionospheric gradient under geomagnetic perturbations were investigated using CORS data from America, Italy, Australia, and New Zealand. The hazard of the ionospheric gradient to GBAS was modelled by the overbound, based on the standard deviation of the Gaussian distribution, and covered all probable ionospheric gradients for the period involved. The variability of overbounds was analyzed for a two-month period in 2017, showing that it varied closely in relation to the geomagnetic perturbations. Some detailed conclusions are listed as follows:

1. The ionospheric gradient decreased greatly from the auroral region to the middle latitudes for all the periods considered, especially during the geomagnetic storm, when the overbound values drastically increased; the maximum ionospheric gradient discovered in the American region approached 980 mm/km at 01:07 UT at the baseline range 25–30 km. The value exceeded the ionospheric gradient discovered at Brazil low latitudes by [15,16]. The large ionospheric gradients were concentrated in the main phase of the geomagnetic storm. The result is probable attributed to the ionospheric irregularities generated by the geomagnetic storm, leading to the spatial ununiform of ionosphere TEC.
2. The ionospheric gradient was dependent on the baseline distance and usually decreased with increasing baseline distance. As a key parameter to indicate GBAS integrity hazard, the overbound value showed similar variability and acted as statistical indicator of ionospheric gradient. The overbound value derived for baseline distances less than 10 km was several times larger than those derived for baseline distances larger than 10 km. It decreased to less than 12 mm/km when the baseline distance exceeded 15 km under geomagnetically quiet conditions, which was consistent with a pervious study conducted in Thailand airport [18]. The standard deviation of the ionospheric gradient stayed below 4 mm/km for all the considered cases on geomagnetically quiet days, which was consistent with previous investigations of the solar cycle 23 [7,8,29].
3. The overbound value exhibited great variabilities corresponding to the baseline distance under the geomagnetic storm. In this storm case, it fluctuated in both the sub-auroral region and the middle latitudes. Two probable reasons were attributed to the following: First, the ionospheric irregularities generated by the storm induced PPEF, and these irregularities were responsible for large ionospheric gradients according to previous studies [12,19,36,37,38,39]; second, the MSTID accompanied the geomagnetic storm, and similar feature has been noticed in Asia during the MSTID on 10 November 2004 [20,30].
4. The overbound values observed during this geomagnetic storm showed hemispherical asymmetry, with the values in America being the most dominant, which showed consistency with the results of [26]. A probable explanation for this correlation is the geographical factor, that is, America covers both the auroral and sub-auroral regions, thereby contributing large values of to the overall results. Moreover, the monthly variability of the overbound values for America, Australia, and New Zealand also indicated the strong impact of geomagnetic perturbations on the formation and magnitude of ionospheric gradients.
5. The standard deviation of the ionospheric gradient during geomagnetically quiet days decreases with increasing baseline distance, within 4 mm/km for the considered cases in this work; while it increases drastically under geomagnetic disturbance, and shows inconsistency when the baseline distance between 15 and 20 km on 7 September, after a X-class solar flare and before a geomagnetic storm on 8 September. Similar features were discovered on the storm day, 8 September, when the baseline distance was 15 to 20 km in the sub-auroral region and the baseline distance was 20 to 25 km in the middle latitudes.

## Figures and Tables

**Table 1:** Dependence of baseline distance for ionospheric gradient during quiet days.

Day	Ionospheric Gradient	Baseline Distance (km)					
		<10	10–15	15–20	20–25	25–30	30–35
220	$\sigma_{\mathrm{overbound}}$ (mm/km)	25.42	4.62	7.38	6.96	4.76	2.91
	$\sigma_{\mathrm{vig}}$ (mm/km)	8.49	2.99	2.73	2.04	1.62	1.25
	f	2.95	1.50	2.55	3.35	2.85	2.30
221	$\sigma_{\mathrm{overbound}}$ (mm/km)	31.62	5.79	9.47	5.90	4.63	4.36
	$\sigma_{\mathrm{vig}}$ (mm/km)	9.19	3.03	2.80	2.07	1.65	1.40
	f	3.40	1.85	3.30	2.75	2.75	3.10
222	$\sigma_{\mathrm{overbound}}$ (mm/km)	53.94	5.09	5.72	9.48	6.51	3.89
	$\sigma_{\mathrm{vig}}$ (mm/km)	9.69	3.03	2.56	2.06	1.68	1.34
	f	5.55	1.60	2.10	4.55	3.85	2.90

**Table 2:** Dependence of baseline distance for ionospheric gradient during the storm days.

Day	Ionospheric Gradient	Baseline Distance(km)					
		<10	10–15	15–20	20–25	25–30	30–35
250	$\sigma_{\mathrm{overbound}}$ (mm/km)	70.98	24.46	47.94	23.36	16.12	10.62
	$\sigma_{\mathrm{vig}}$ (mm/km)	15.37	5.64	6.79	4.27	3.16	2.68
	f	4.50	4.25	7.00	5.40	5.00	3.85
251	$\sigma_{\mathrm{overbound}}$ (mm/km)	325.31	66.61	63.02	39.50	58.61	38.63
	$\sigma_{\mathrm{vig}}$ (mm/km)	50.41	12.53	7.85	7.15	5.26	4.56
	f	6.45	5.25	8.00	5.45	11.15	8.45

**Table 3:** Regional behaviors of the overbound values and the standard deviation during the quiet days.

Day	Regions		<10 km	10–15 km	15–20 km	20–25 km	25–30 km	30–35 km
220	Auroral	$\sigma_{\mathrm{overbound}}$ (mm/km)	29.68	—	—	—	—	—
		$\sigma_{\mathrm{vig}}$ (mm/km)	9.81	—	—	—	—	—
	Sub-Auroral	$\sigma_{\mathrm{overbound}}$ (mm/km)	28.61	19.70	7.78	7.56	5.03	1.72
		$\sigma_{\mathrm{vig}}$ (mm/km)	6.44	2.70	2.42	1.80	1.21	0.96
	Middle Latitudes	$\sigma_{\mathrm{overbound}}$ (mm/km)	18.34	5.08	5.84	4.05	4.75	3.06
		$\sigma_{\mathrm{vig}}$ (mm/km)	8.66	3.17	2.94	2.15	1.93	1.48
221	Auroral	$\sigma_{\mathrm{overbound}}$ (mm/km)	37.89	—	—	—	—	—
		$\sigma_{\mathrm{vig}}$ (mm/km)	16.18	—	—	—	—	—
	Sub-Auroral	$\sigma_{\mathrm{overbound}}$ (mm/km)	18.39	18.38	10.05	4.89	3.11	2.48
		$\sigma_{\mathrm{vig}}$ (mm/km)	5.00	2.60	2.55	1.65	1.16	1.03
	Middle Latitudes	$\sigma_{\mathrm{overbound}}$ (mm/km)	18.38	5.50	8.13	6.13	4.90	4.59
		$\sigma_{\mathrm{vig}}$ (mm/km)	7.78	3.22	2.99	2.24	2.00	1.66
222	Auroral	$\sigma_{\mathrm{overbound}}$ (mm/km)	70.32	—	—	—	—	—
		$\sigma_{\mathrm{vig}}$ (mm/km)	17.09	—	—	—	—	—
	Sub-Auroral	$\sigma_{\mathrm{overbound}}$ (mm/km)	36.81	16.64	4.08	10.49	7.00	2.50
		$\sigma_{\mathrm{vig}}$ (mm/km)	6.47	2.64	1.83	1.70	1.23	1.07
	Middle Latitudes	$\sigma_{\mathrm{overbound}}$ (mm/km)	13.38	5.37	5.98	5.27	4.05	4.23
		$\sigma_{\mathrm{vig}}$ (mm/km)	7.76	3.19	2.99	2.18	1.95	1.54

**Table 4:** Regional behaviors of the overbound values and the standard deviation during the storm.

Day	Region		<10 km	10–15 km	15–20 km	20–25 km	25–30 km	30–35 km
250	Auroral	$\sigma_{\mathrm{overbound}}$ (mm/km)	83.67	—	—	—	—	—
		$\sigma_{\mathrm{vig}}$ (mm/km)	28.8	—	—	—	—	—
	Sub-Auroral	$\sigma_{\mathrm{overbound}}$ (mm/km)	23.41	26.07	50.98	23.99	15.42	11.01
		$\sigma_{\mathrm{vig}}$ (mm/km)	7.25	6.97	8.44	3.75	3.40	2.84
	Middle Latitudes	$\sigma_{\mathrm{overbound}}$ (mm/km)	25.64	8.16	7.48	19.94	15.82	10.85
		$\sigma_{\mathrm{vig}}$ (mm/km)	11.11	3.54	3.94	3.20	2.54	2.53
251	Auroral	$\sigma_{\mathrm{overbound}}$ (mm/km)	365.64	—	—	—	—	—
		$\sigma_{\mathrm{vig}}$ (mm/km)	107.48	—	—	—	—	—
	Sub-Auroral	$\sigma_{\mathrm{overbound}}$ (mm/km)	70.70	69.13	65.79	31.80	60.69	33.88
		$\sigma_{\mathrm{vig}}$ (mm/km)	15.36	9.59	9.73	5.25	5.96	4.61
	Middle Latitudes	$\sigma_{\mathrm{overbound}}$ (mm/km)	87.53	32.78	21.68	29.84	22.69	40.29
		$\sigma_{\mathrm{vig}}$ (mm/km)	15.28	4.97	4.87	5.00	3.41	4.38

**Table 5:** Regional behaviors of the overbound values and the standard deviation during the quiet days.

Region		220	221	250	251
USA	$\sigma_{\mathrm{overbound}}$ (mm/km)	21.28	17.81	61.18	148.20
	$\sigma_{\mathrm{vig}}$ (mm/km)	4.57	4.31	9.22	19.12
Italy	$\sigma_{\mathrm{overbound}}$ (mm/km)	12.78	14.40	17.79	49.02
	$\sigma_{\mathrm{vig}}$ (mm/km)	5.57	6.06	5.79	6.60
Australia	$\sigma_{\mathrm{overbound}}$ (mm/km)	35.17	16.52	6.96	38.95
	$\sigma_{\mathrm{vig}}$ (mm/km)	4.55	6.11	3.36	11.76
New Zealand	$\sigma_{\mathrm{overbound}}$ (mm/km)	9.68	8.75	10.05	20.79
	$\sigma_{\mathrm{vig}}$ (mm/km)	3.34	3.06	3.49	4.17
